# Antibiotic Susceptibility of *β*-Glucuronidase-Positive *Escherichia coli* Isolated from Poultry Products in Morocco

**DOI:** 10.1155/2023/7862168

**Published:** 2023-04-03

**Authors:** Oumaima Fazza, Mireille Favard Ennachachibi, Houda Ennassiri, Abdelaziz Hmyene

**Affiliations:** ^1^Laboratory of Biochemistry, Environment and Agri-Food, Faculty of Sciences and Technology Mohammedia, University Hassan II, Casablanca, Morocco; ^2^Charles Nicolle Laboratory of Medical Analysis, Casablanca, Morocco; ^3^Charles Nicolle Laboratory of Environment and Food Safety, Casablanca, Morocco

## Abstract

Poultry products are one of the main foodstuffs consumed in Morocco. The deterioration of their hygienic quality and the emergence of antibiotic resistance are the major public health problems. This study was carried out to determine the level of emergence of antibiotic resistance. For that, between May 2021 and June 2022, poultry products (e.g., minced meat, sausage, and meat) were collected aseptically in sterile bags from retail stores in different cities of Morocco, labeled, and transported in chilled conditions to the laboratory. The bacterial analysis was performed within 24 hours. Isolation and identification of *Escherichia coli* were performed according to the Moroccan standard NM ISO 16649 2018. Antimicrobial susceptibility determination of 23 antibiotics was tested using the Kirby-Bauer disk diffusion method. The results showed a high level of antimicrobial resistance to amoxicillin (58%), amoxicillin-clavulanic acid (54%), piperacillin (63%), trimethoprim (32%), nalidixic acid (46%), and ofloxacin (43%). 58 isolates (60%) were positive for beta-lactamase class A (penicillinase) test detection, and 2 isolates (3%) produced extended-spectrum beta-lactamases. The high level of resistance demonstrated in this study should alert health organizations in the country. An establishment of surveillance programs to control the use and the administration of antibiotics in the poultry field and initiation of reliable methods to follow up on the latest updates on the emergence of bacterial resistance is a necessity.

## 1. Introduction

*Escherichia coli* is a Gram-negative, facultative anaerobic, rod-shaped bacterium of the genus *Escherichia*; it is mostly harmless. In addition to its significant role as a part of the intestinal flora of most animal species, as well as humans, it is considered a bioindicator of hygiene and antimicrobial resistance [1]. However, according to the World Health Organization, some serotypes can be life-threatening, such as Shiga toxin-producing *E. coli* (STEC) [2].

*E. coli* is considered one of the most common foodborne pathogens [3]; the consummation admin of food contaminated with certain isolates of *E. coli* can cause severe complications such as urinary tract infections, respiratory illness, and pneumonia.

Bacteria have different mechanisms of resistance to antibiotics: inactivation of these agents by the production of beta-lactamases changes the target protein in the cell wall, reduces the permeability of the outer membrane, and increases the expression of drug efflux pump.

Gram-negative bacteria have evolved the production of various *β*-lactamases; in the case of *E. coli*, the extended-spectrum *β*-lactamases (ESBLs) and AmpC *β*-lactamases (AmpC) are the most important enzymes in the medical field; these enzymes present different spectrum of hydrolytic activity.

ESBLs are generally plasmid encoded; strains producing the TEM and SHV types were the first discovered. However, since two decades, ESBLs of CTX-M type are the predominant ESBL enzymes both in humans and animals [4]. Contrary to the SHV and TEM types, CTX-M groups seem to have originated from the chromosomally encoded ESBL genes from different Kluyvera species [4].

AmpC was originally described as a chromosomally encoded enzyme. However, resistance can be plasmid encoded too. In the case of *E. coli*, dissemination of the AmpC gene is initiated by the horizontal transfer of plasmids; this phenomenon leads to the occurrence of AmpC resistance in bacteria that previously lacked or expressed low levels of these genes [4].

The animals infected with antimicrobial-resistant bacteria spread the resistance to the humans through the environment via excreta, food products, and direct contact. The foodborne route probably is the most important [5]. The transmission of the antimicrobial resistance causes a great harm to human health and to the breeding industry.

Poultry products are considered the main carrier of pathogenic bacteria such as *E. coli*. The higher prevalence of antibiotic-resistant *E. coli* was recorded in poultry meat more than other kinds of meat [6, 7].

The emergence of antibiotic resistance among *E. coli* is a major public health problem nowadays. The misuse of antibiotics in poultry fields and the absence of severe and strict legislation especially in low- and lower-middle-income countries are the major factors of this emergence.

The aim of this study was to determine the level of contamination with beta-glucuronidase-positive *E. coli*, to determine the prevalence of antimicrobial-resistant *E. coli* in poultry products from different cities in Morocco, and to understand its public health significance. Moreover, the prevalence of ESBL/AmpC-producing *E. coli* isolate was investigated.

## 2. Materials and Methods

### 2.1. Sample Collection

Between May 2021 and June 2022, poultry products (*n* = 154), minced meat (*n* = 40), merguez (*n* = 31), chicken gizzard (*n* = 4), sausage (*n* = 16), mechanically separated meat (*n* = 15), meat cuts (*n* = 35), whole chicken (*n* = 7), and fish meal (*n* = 6) were collected aseptically in sterile bags from retail stores in different cities of Morocco (Casablanca, Marrakech, Rabat, Berrechid, Benslimane, Tangier, Tetouan, El Hajeb, Salé, El Jadida, Meknes, Fez, Nador, Immouzar, Temara, Berkane, and Oujda), labeled, and transported in chilled conditions to the laboratory. Analysis was performed within 24 hours.

### 2.2. Isolation of Bacteria

The isolation and the identification of *E. coli* were performed according to the Moroccan standard NM ISO 16649 2018 microbiology of the food chain, horizontal method for the enumeration of beta-glucuronidase-positive *Escherichia coli*.

Approximately 10 g of the sample was placed in a sterile filtered stomacher bag, and then, 90 mL of Buffered Peptone Water (BPW) (Oxoid, CM0509B) was added. Dilutions were prepared using a gravimetric dilutor (Interscience, DiluFlow 3 kg, 501103), and the suspension was grinded until the dispersion of clumps.

Using a sterile plastic serological pipette, 1 mL of each initial suspension (10^−1^) was transferred to a sterilized Petri dish, and approximately 15 mL of the Tryptone Bile X-glucuronide medium (TBX) was added (Tryptone Bile X-glucuronide agar, Biokar Diagnostics, BK130HA).

The inoculums were rigorously mixed to the medium and put down to solidify by placing the Petri dishes on a horizontal surface for 15 min; the Petri dishes were upturned and incubated at 44°C for 18 to 24 hours.

After the incubation period, one of the blue colonies was isolated and confirmed by an indole test using tryptophan broth (Biokar Diagnostics, BK163HA); the appearance of a cherry-red ring on the top of the medium within a few seconds confirms the ability of the microorganism isolated to degrade tryptophan and produce indole.

A second biochemical confirmation was carried out by API 20E (bioMérieux SA, 20100).

### 2.3. Susceptibility to Antibiotics

Ninety-six isolates (96) of *E. coli* were examined for susceptibility of twenty-three (23) antimicrobials.

Antimicrobial susceptibility was tested using the Kirby-Bauer disk diffusion method [8].

As described in Table 1, the following antibiotic disks were applied on Mueller-Hinton agar: amikacin (AK), amoxicillin-clavulanic acid (AMC), ampicillin (AM), azithromycin (AZM), aztreonam (ATM), cefadroxil (CFR), cefepime (FEP), cefotaxime (CTX), cefoxitin (FOX), ceftazidime (CAZ), chloramphenicol (C), ciprofloxacin (CIP), colistin (CT), trimethoprim-sulfamethoxazole (SXT), levofloxacin (LEV), ofloxacin (OFX), piperacilline (PRL), piperacilline tazobactam (TPZ), tobramycin (TOB), trimethoprim (TMP), gentamicin (CN), and tigecycline (TGC).

The inhibition diameter of each antibiotic was determined using a vernier caliper and represented in millimeters. Interpretation of the results was performed according to the 2021 guidelines of the European Committee on Antimicrobial Susceptibility Testing (EUCAST) [9] and the 2021 veterinary guidelines of the Antibiogram Committee of the French Microbiology Society (CA-SFM) [10].

### 2.4. Detection of Penicillinase or Beta-Lactamase Class A

The isolates that showed resistance to penicillins (ampicillin and piperacillin) and susceptibility to the first generation of cephalosporins (cefadroxil) were considered penicillinase-producing isolates.

### 2.5. Detection of Extended-Spectrum Beta-Lactamase (ESBL)

Extended-spectrum beta-lactamase (ESBL) phenotype was detected by demonstrating the effect of clavulanic acid on the third or fourth generation of cephalosporin's antibiotics (cefotaxime and cefepime).

The qualitative method consists of using synergy test between two disks on the standard antibiogram. A disk of the third or fourth generation of cephalosporin's antibiotic and a disk containing clavulanic acid (e.g., amoxicillin-clavulanic acid) are placed 30 mm apart [9]. If there is synergy between the clavulanic acid and the C3G/C4G, the diameter of the inhibition zone between the two disks increases and a characteristic image of synergistic action referred to as "champagne cork" [10]. Therefore, the bacterium is considered an ESBL producer.

### 2.6. Detection of High Level of Cephalosporinase Producing

The utilization of cefoxitin in antibiograms makes it possible to distinguish between isolates with ESBL or with high-level production of cephalosporinase.

Isolates with high-level production of cephalosporinase are resistant to cefoxitin; meanwhile, ESBL isolates are susceptible [10].

### 2.7. Statistical Analysis

Percentage of resistance to each antibiotic was determined and illustrated into a graph by Microsoft Excel 2007.

## 3. Results

In the study, out of the total 154 samples analyzed, 62.33% (*n* = 96) were contaminated with *E. coli*. Percentages of positive simples were 75% (minced meat), 58% (merguez), 75% (chicken gizzard), 50% (sausage8), 60% (mechanically separated meat9), 68.5% (meat cuts), 28.5% (whole chicken), and 33.3% (fish meal). The highest percentages of positive samples were detected in Berrechid, Casablanca, Rabat, and Tangier with 23.9%, 12.5%, 11.4%, and 10.4%, respectively.

Table 2 illustrates the results of the antibiotic susceptibility of the isolates studied. Numbers of resistant/intermediate isolates were mentioned.

**Figure 1:**
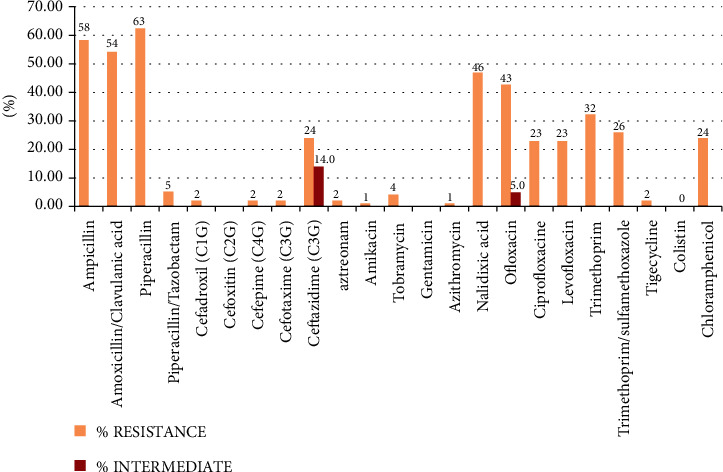
Profile of the antimicrobial resistance in the 96 *E. coli* isolates.

As illustrated in Figure 1, the antibiograms showed high resistance to ampicillin (58.3%), amoxicillin/clavulanic acid (54.1%), piperacillin (62.5%), trimethoprim (32.2%), nalidixic acid (46.8%), and ofloxacin (42.7%).

In total of 96 *E. coli* isolates, 60% were positive to the test of detection of penicillinase or beta-lactamase class A; the isolates were able to produce penicillinase, an enzyme that disrupt the antimicrobial effect of penicillins.

**Figure 2:**
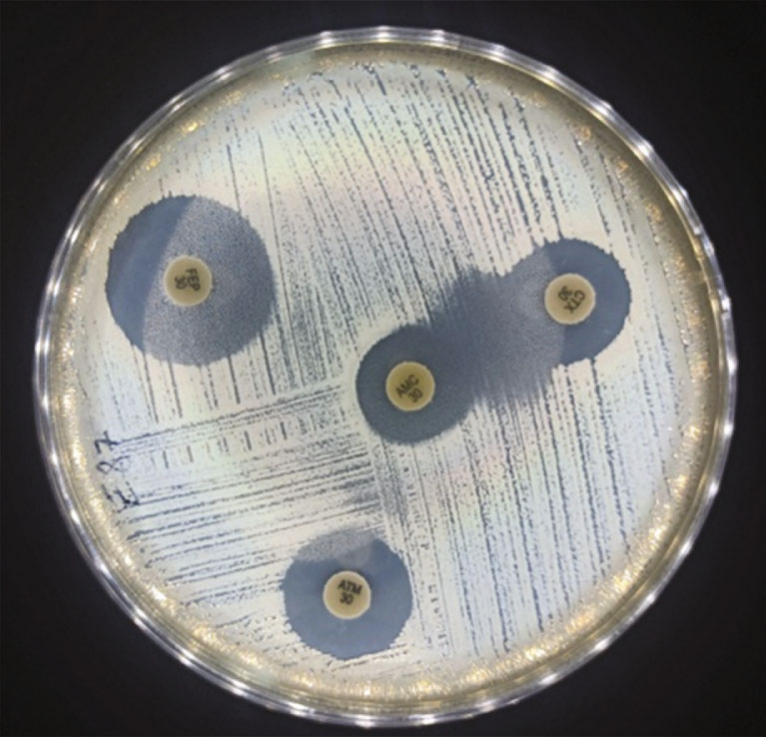
Synergy form between amoxicillin/clavulanic acid and cefotaxime.

In addition, 2 isolates (2%) were positive to the test of synergy between the clavulanic acid and the C3G; the isolates had a high level of resistance to penicillins and the first and third generations of cephalosporin and azithromycin; Figure 2 presents the image of synergistic action obtained.

No high level of cephalosporinase producing was detected; indeed, the 96 isolates studied were susceptible to cefoxitin.

## 4. Discussion

Poultry is one of the main foodstuffs consumed in the country, and it is considered one of the first reservoir of *E. coli*. The monitoring of the microbial contamination of these foodstuffs should be a crucial occupation for health minister and food industry professionals.

In fact, according to Cohen et al. [11], 48.4% of the analyzed samples tested positive for *E. coli*, 52% samples of turkey, and 43% samples of chicken. Furthermore, in 2018, according to Hassna et al. [12], 49% of the samples were positive to *E. coli*. These results reflect the state of hygiene of this type of foodstuffs.

In this study, 62.3% of samples were *E. coli* positive. Minced meat, chicken gizzard, and meat cut samples were the most contaminated with *E. coli* (75%, 75%, and 69%, respectively), followed by merguez, sausage, whole chicken, and fish meal samples (58%, 60%, 29%, and 33%, respectively). Most of the positive samples were detected in the northwest of the country, since this region provides 45% of the national avian meat production [13].

These results confirm the results of the previous studies. Poultry products showed a remarkable level of contamination with *E. coli*. This hygienic state of poultry products may be due to the poor conditions of the raw material or to contaminations in the food chain.

The susceptibility test results showed a remarkable level of antimicrobial resistance to the first-line antibiotic treatments: aminopenicillins without beta-lactamase inhibitors (58.3%), chloramphenicol (23.9%), trimethoprim (32.2%), and trimethoprim-sulfamethoxazole (26%). An overall increase in ampicillin antimicrobial resistance was found in this study, in comparison to a study performed earlier in 2018 on isolates from poultry farmers and poultry slaughters (45.45% and 38%, respectively) [14]. High level of resistance to ampicillin was detected in Saudi Arabia and the United States of America (78.4% and 62%, respectively) [15, 16].

However, to trimethoprim-sulfamethoxazole and chloramphenicol, a lower level of resistance was found in comparison with studies performed in 1888, 1995, and 2015 in Morocco [17–19]. In fact, the resistance to sulfonamides, dihydrofolate reductase inhibitors, and combinations in Morocco is mainly due to the extensive use of the combination trimethoprim/sulfamethoxazole as a prevention factor against omphalitis in chicks and to control avian colibacillosis and salmonellosis. Trimethoprim/sulfamethoxazole is wildly used due to its low cost to conquer the resistance developed toward tetracyclines and sulfonamides [12].

Studies carried out in different countries showed a higher resistance to trimethoprim-sulfamethoxazole, 70.2% in Algeria [20], 80% in Tunisia, and 84% in Bangladesh [21, 22]. Moreover, to chloramphenicol, lower level of resistance was detected in Algeria and India with the percentages of 10.5% and 8%, respectively [20, 23].

These substances belong to the category D of antibiotics according to the Antimicrobial Advice Ad Hoc Expert (AMEG) Categorization [24]. The antibiotics in this category present the lowest risk to public health; however, some of them are listed by WHO as critically important antimicrobials for human medicine CIAs (ampicillin and amoxicillin) [25]. Therefore, unnecessary administration and unnecessarily long treatment periods should be avoided.

Moreover, the administration of chloramphenicol is prohibited to any animal whose meat or products are intended for human consumption in Morocco [26]. Authorities had established a surveillance program for some antimicrobials used in poultry such as chloramphenicol and gentamicin. However, these programs remain insufficient for the fact that they do not perform monitoring for antimicrobial use and consumption in food animals. Furthermore, there were no reported studies concerning the use and consumption of antimicrobials in the broiler production in Morocco [27]. Indeed, resistance to chloramphenicol might be due to an illicit use of this antimicrobial or the presence of a transferable plasmid that confers multiple-drug resistance such as Inc plasmids [28].

Level of resistance to amoxicillin-clavulanic acid was 42.42% in 2018 [14]; the results of this study showed an increase up to 54.1%. Other results showed divergent percentages of resistance, 92.1% in Algeria and 4% in Tunisia [20, 21].

Aminopenicillin in combination with beta-lactamase inhibitor is defined as a category C antibiotic. The antibiotics classified in this category have a higher risk of antimicrobial resistance to human and/or animal health than antibiotics classified in category D, according to AMEG.

Ureidopenicillins alone (e.g., piperacillin) or in combination with beta-lactamase inhibitors such as tazobactam are nonauthorized for veterinary medicine in the European Union. Moreover, they are classified as critical antimicrobials for human medicine. However, the results illustrate a high percentage of resistance to piperacillin (62.5%), which reveals the level of maltreatment of this antibiotic in the poultry field. The results showed a low resistance to the combination of piperacillin-tazobactam (5.2%).

Resistance to nalidixic acid, ofloxacin, and levofloxacin was observed in 46.8%, 42.7%, and 22.9% isolates, respectively. A remarkable increase in the level of resistance to nalidixic acid in comparison with the results was obtained in 1988 and 1995 (4% and 25%, respectively). A remarkable percentage of resistance was detected in Tunisia and Saudi Arabia (67% and 70.3%, respectively) [15, 21]. These antibiotics, belonging to quinolones/fluoroquinolones, are critically important for human medicine for the reason that few therapeutic alternative options exist.

In addition, the high sensitivity of the isolates to azithromycin, amino-glycosides, tetracyclines, macrolides, and colistin confirms the results of other studies. This low level of resistance reflects the low or moderate use of these drugs in poultry therapy due mostly to their inactivity on systemic colibacillosis.

No previous studies had described the prevalence and the dominance of genes related to ESBL *E. coli* isolated from poultry or bovine products. However, according to Nayme et al. [29], out of 144 *E. coli* isolates detected in vegetable salads, four were ESBL producers. blaCTX-M14 was the only gene detected [29]. Many studies had revealed the correlation between food contaminated with drug-resistant bacteria and the emergence of ESBL genes in the bacterial flora of humans; in Morocco, the blaSHV-12 and blaCTX-M-15 were the most frequent ESBL genes detected in fecal samples recovered from the community [30]. From another perspective, blaCTX-M-15 was the most frequent ESBL *E. coli* genotype detected in urinary sample isolates from different Moroccan cities [31].

## 5. Conclusion

The high level of resistance revealed should alert health organizations in the country. Surveillance programs should be established to follow up the latest update on the emergence of bacterial resistance in the country. Moreover, the control of the use and the administration of antibiotics in animal therapy and precisely in the poultry field is a must.

## Figures and Tables

**Table 1:** List of the antibiotics selected and their potency.

Antibiotic	Disk potency (*μ*g)	Classification
Ampicillin	10	*β*-Lactams
Amoxicillin-clavulanic acid	20-10	
Piperacillin	30	
Piperacillin/tazobactam	30-6	
Aztreonam	30	

Cefadroxil	30	1^st^-generation cephalosporin
Cefoxitin	30	2^nd^-generation cephalosporin
Ceftazidime	10	3^th^-generation cephalosporin
Cefotaxime	5	3^th^-generation cephalosporin
Cefepime	30	4^th^-generation cephalosporin

Amikacin	30	Amino-glycosides
Tobramycin	10	
Gentamicin	10	

Nalidixic acid	30	1^st^-generation quinolone
Ofloxacin	5	2^nd^-generation quinolone
Levofloxacin	5	3^th^-generation quinolone

Azithromycin	15	Macrolides

Trimethoprim	5	DHFR inhibitors
Trimethoprim-sulfamethoxazole	1.25-23.75	

Colistin	50	Polymyxin

Chloramphenicol	30	Phenicols

Tigecycline	15	Tetracyclines

**Table 2:** Number of *E. coli* isolates resistant/intermediate for each tested antibiotic.

Antibiotics	Resistant isolates	Intermediate isolates
Ampicillin	56	—
Amoxicillin/clavulanic acid	52	—
Piperacillin	60	—
Piperacillin/tazobactam	5	—
Cefadroxil (C1G)	2	—
Cefoxitin (C2G)	0	0
Cefepime (C4G)	2	0
Cefotaxime (C3G)	2	0
Ceftazidime (C3G)	23	13
Aztreonam	2	0
Amikacin	1	—
Tobramycin	4	—
Gentamicin	0	—
Azithromycin	1	—
Nalidixic acid	45	—
Ofloxacin	41	5
Ciprofloxacin	11	—
Levofloxacin	22	5
Trimethoprim	31	—
Trimethoprim/sulfamethoxazole	25	0
Tigecycline	2	—
Colistin	0	—
Chloramphenicol	23	—

## Data Availability

The authors confirm that the data supporting the findings of this study are available within the article. Raw data that support the findings are available from the corresponding author, upon reasonable request.
